# Molecular insights into AGS3’s role in spindle orientation: a biochemical perspective

**DOI:** 10.1093/jmcb/mjae049

**Published:** 2024-11-23

**Authors:** Shi Yu, Jie Ji, Jingwei Weng, Zhijun Liu, Wenning Wang

**Affiliations:** Department of Chemistry, Institute of Biomedical Sciences and Multiscale Research Institute of Complex Systems, Fudan University, Shanghai 200438, China; Department of Chemistry, Institute of Biomedical Sciences and Multiscale Research Institute of Complex Systems, Fudan University, Shanghai 200438, China; Department of Chemistry, Institute of Biomedical Sciences and Multiscale Research Institute of Complex Systems, Fudan University, Shanghai 200438, China; National Facility for Protein Science in Shanghai, Zhangjiang Lab, Shanghai Advanced Research Institute, Chinese Academy of Sciences, Shanghai 201210, China; Department of Chemistry, Institute of Biomedical Sciences and Multiscale Research Institute of Complex Systems, Fudan University, Shanghai 200438, China

**Keywords:** asymmetric cell division, spindle orientation, AGS3, LGN, NuMA

## Abstract

The intrinsic regulation of spindle orientation during asymmetric cell division depends on the evolutionarily conserved protein complex LGN (Pins)/NuMA (Mud)/Gα⋅GDP. While the role of LGN and its *Drosophila* orthologue Pins is well-established, the function of AGS3, the paralogue of LGN, in spindle orientation during cell division remains controversial. This study substantiates the contentious nature of AGS3’s function through systematic biochemical characterizations. The results confirm the high conservation of AGS3 in its functional structural domains, similar to LGN, and its comparable ability to bind to partners including NuMA, Insc, and Gα_i3_⋅GDP. However, in contrast to LGN, AGS3 and the microtubule-binding protein NuMA are unable to form stable hetero-hexamers or higher-order oligomeric complexes that are pivotal for effective regulation of spindle orientation. It was found that this notable difference between AGS3 and LGN stems from the N-terminal sequence preceding the conserved TPR motifs, which spans ∼20 residues. Furthermore, our findings substantiate the disruptive effect of Insc on the oligomeric AGS3/NuMA complex, while showing no impact on the oligomeric LGN/NuMA complex. Consequently, Insc emerges as an additional regulatory factor that distinguishes the functional roles of AGS3 and LGN, leading to the impairment of AGS3’s ability to actively reorient the mitotic spindle. These results elucidate the molecular basis underlying the observed functional disparity in spindle orientation between LGN and AGS3, providing valuable insights into the regulation of cell division at the molecular level.

## Introduction

In multicellular organisms, asymmetric cell division (ACD) is a fundamental process essential for the generation of diverse cell types (Morrison and Kimble, 2006; Konno et al., 2008a). During ACD, the correct alignment of the mitotic spindle plays a critical role in ensuring the unequal segregation of cell fate determinants and production of daughter cells of varying sizes. This process is tightly regulated by a variety of factors, including intrinsic signals and extrinsic cues, as well as mechanical forces (Wu et al., 2008; Lechler and Mapelli, 2021). In the intrinsic signaling regulation of mitotic spindle orientation across various cell types, the protein LGN (Pins in *Drosophila*) plays a pivotal role (Du and Macara, 2004). LGN connects with the apical polarity complex Par3/Par6/aPKC through interactions with Inscuteable (Insc) (Izaki et al., 2006; Kamakura et al., 2013; Williams et al., 2014; Yan et al., 2022) and forms the conserved complex LGN/NuMA/Gα_i_. In this complex, the microtubule-binding protein NuMA interacts with the motor protein dynein to exert force on astral microtubules, thereby reorienting the spindle (Kiyomitsu and Cheeseman, 2013; Zheng et al., 2013).

**Figure 1:**
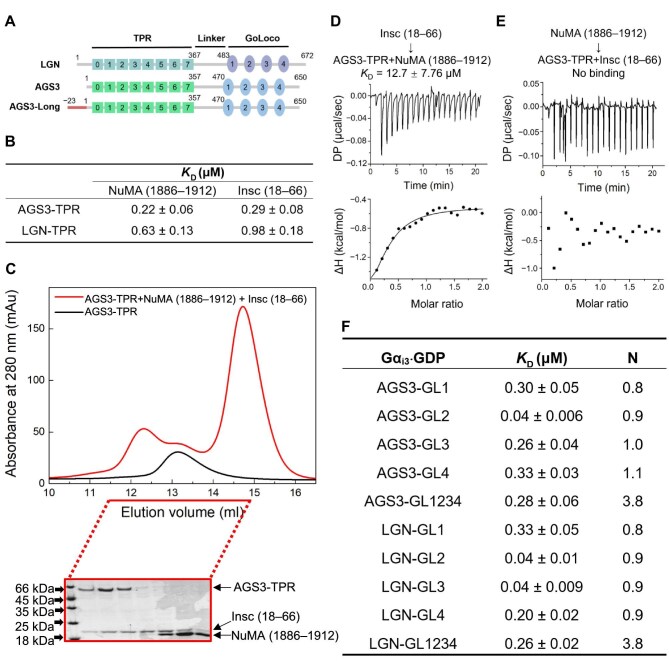
AGS3-TPR and AGS3-GL interact with their partners similarly to LGN. (**A**) The domain organizations of LGN and AGS3 isoforms. (**B**) ITC-based binding affinities of the TPR domains of AGS3 and LGN to Insc and NuMA. The raw data and fits are shown in Supplementary Figure S1. (**C**) SEC analysis of the mixture of AGS3-TPR, NuMA (1886–1912), and Insc (18–66) at a 1:2:2 ratio. (**D**) ITC measurement of the binding of Insc (18–66) to the mixture of AGS3-TPR and NuMA (1886–1912). (**E**) ITC measurement of the binding of NuMA (1886–1912) to the mixture of AGS3-TPR and Insc (18–66). (**F**) ITC-based binding affinities of Gα_i3_⋅GDP to the GL motifs and GoLoco domains of AGS3 and LGN. The raw data and fits are shown in Supplementary Figure S2.

Unlike Pins in *Drosophila*, vertebrates possess the paralogues LGN and AGS3, both of which belong to the type II class of activator of G-protein signaling (AGS) family, functioning in G-protein signaling activation independently of receptors (Bernard et al., 2001; Blumer et al., 2012). AGS3 shares high sequence similarity with LGN, characterized by an N-terminal tetratricopeptide repeat (TPR) domain containing eight TPR motifs and a C-terminal GoLoco domain containing four GoLoco (GL) motifs (Figure 1A; Groves et al., 2007; Jia et al., 2012). In addition to its involvement in G protein-coupled receptor signaling regulation, AGS3 has been linked to various cellular functions, including neuronal differentiation, autophagy, protein transport, and brain adaptation mechanisms associated with the renal injury response (Pattingre et al., 2003; Groves et al., 2007; Blumer et al., 2008; Bowers et al., 2008; Fan et al., 2009; Regner et al., 2011).

Nonetheless, the role of AGS3 in spindle orientation remains controversial. Early research suggested that AGS3 perturbs planar spindle orientation in apical divisions of neuronal progenitor cells (Sanada and Tsai, 2005). However, another study observing RNAi targeting AGS3 did not reveal a more planar spindle orientation phenotype (Konno et al., 2008b). Subsequently, Saadaoui et al. (2017) discovered that, despite its conserved interactions with NuMA and Gα_i_  *in vitro*, AGS3 cannot substitute LGN for spindle orientation function *in vivo*. More recently, Descovich et al. (2023) found that AGS3 promotes planar divisions by counteracting LGN's ability in promoting and maintaining vertical divisions, implying opposing roles for these two vertebrate paralogues in regulating epidermal spindle orientation and differentiation during development. These observations underscore the need to understand the structural foundation underlying the distinct functional roles of LGN and AGS3 in spindle orientation. While some studies have investigated AGS3’s domain interactions (Bernard et al., 2001; Adhikari and Sprang, 2003; Sanada and Tsai, 2005; Saadaoui et al., 2017; Wavreil and Yajima, 2020; Yip et al., 2020), a comprehensive comparison of LGN and AGS3 in terms of their domain structures and protein–protein interactions, especially with quantitative evaluation, remains lacking.

In this study, we conducted a comprehensive biochemical characterization to parallelly investigate the inter- and intramolecular interactions of AGS3 and LGN. Our findings indicate that the binding affinities of the TPR domain to NuMA and Insc, as well as the GoLoco domain to Gα_i3_⋅GDP, are very similar between AGS3 and LGN. Additionally, we compared the intramolecular interactions and the opening of the auto-inhibited conformation of AGS3 and LGN, revealing analogous behaviors in both proteins. Furthermore, we evaluated the capabilities of AGS3 and LGN to form hetero-oligomer complexes with NuMA, revealing that the multimeric AGS3/NuMA complex exhibits reduced stability compared to the LGN/NuMA complex. Moreover, the AGS3/NuMA complex is susceptible to disruption by Insc, highlighting a potential regulatory mechanism that differs from LGN/NuMA interactions. These findings provide valuable insights into the molecular mechanisms underlying the functional disparities observed in spindle orientation between AGS3 and LGN. By elucidating these differences at the biochemical level, our study contributes to a better understanding of how these paralogues exert distinct roles in regulating cellular processes, particularly in the context of spindle orientation and ACD.

## Results

### TPR and GoLoco domains of AGS3 display binding patterns similar to those of LGN with their respective partners

Previous studies have demonstrated that the TPR domain of AGS3 can interact with NuMA and Insc similar to LGN (Izaki et al., 2006; Yuzawa et al., 2011; Saadaoui et al., 2017). However, there is a lack of systematic quantification of these interactions. The interactions of AGS3-TPR (1–405) with NuMA and Insc were initially characterized using isothermal titration calorimetry (ITC). The *K*_D_ values for the binding of AGS3-TPR with Insc (18–66) and NuMA (1886–1912) were determined to be 0.29 μM and 0.22 μM, respectively, analogous to those of LGN-TPR (1–409) (Figure 1B; Supplementary Figure S1A–D). Therefore, the TPR domain of AGS3 exhibits comparable affinities for NuMA and Insc as LGN. The mutual exclusivity of NuMA and Insc binding to LGN-TPR has been well-documented, with Insc having the capability to supplant NuMA in the binding process (Culurgioni et al., 2011; Zhu et al., 2011). Here, we examined whether AGS3-TPR also exhibits a preference for binding to Insc over NuMA. To address this, we mixed AGS3-TPR with Insc (18–66) and NuMA (1886–1912) at a 1:2:2 molar ratio and characterized the mixture using size exclusion chromatography (SEC) and sodium dodecyl sulphate–polyacrylamide gel electrophoresis (SDS–PAGE). The results showed that only Insc (18–66) was present in the complex with AGS3-TPR (Figure 1C), suggesting a preferential binding of AGS3-TPR to Insc over NuMA. A similar analysis of the LGN-TPR/Insc/NuMA mixture yielded consistent results with the previous study, showing that LGN preferentially binds to Insc (Supplementary Figure S1E). Additionally, ITC measurements demonstrated that Insc could bind to the 1:1 mixture of AGS3-TPR and NuMA, whereas NuMA could not bind to the 1:1 mixture of AGS3-TPR and Insc (Figure 1D and E). Taken together, these results indicate that the TPR domain of AGS3 shares similar characteristics of partner interactions with LGN-TPR.

Similar to LGN-GoLoco, the GoLoco domain of AGS3 has been demonstrated to interact with Gα_i3_⋅GDP or Gα_i1_⋅GDP (Bernard et al., 2001; Adhikari and Sprang, 2003; Sanada and Tsai, 2005; Yip et al., 2020). Detailed biochemical analyses of the interactions between AGS3-GoLoco and Gα_i1_⋅GDP have revealed binding affinities that resemble those observed between LGN-GoLoco and Gα_i_⋅GDP (Adhikari and Sprang, 2003; Jia et al., 2012). In this study, we specifically investigated the binding affinities of AGS3-Goloco to Gα_i3_⋅GDP. The results showed that each GL motif (∼34 residues) of AGS3 exhibited robust binding to Gα_i3_⋅GDP, displaying binding affinities comparable to those observed for LGN's GL motifs (Figure 1F; Supplementary Figure S2A–H). Furthermore, the full-length GoLoco domains (GL1234) of AGS3 and LGN also showed similar binding affinities to Gα_i3_⋅GDP, consistent with a 1:4 binding stoichiometry (Figure 1F; Supplementary Figure S2I and J). Therefore, both AGS3- and LGN-GoLoco domains can bind four molecules of Gα_i3_⋅GDP simultaneously, with apparent binding affinities resembling to those observed for individual GL motifs (Figure 1F).

### AGS3 forms auto-inhibited conformation through intramolecular interactions

**Figure 2:**
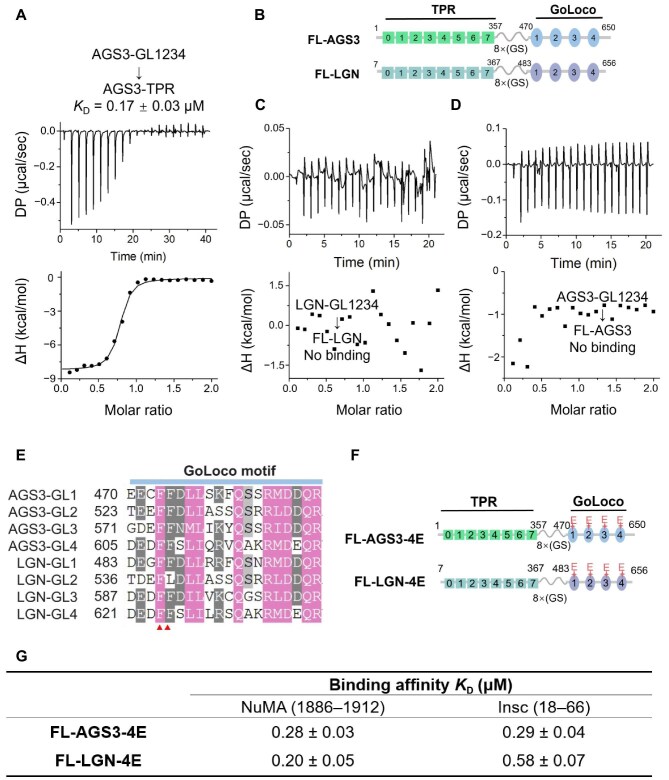
Intramolecular interactions and auto-inhibited conformation of AGS3. (**A**) ITC measurement of the interaction between AGS3-TPR and AGS3-GL1234. (**B**) Schematic diagram of the domain organizations of FL-AGS3 and FL-LGN. (**C**) ITC measurement of the interaction between AGS3-GL1234 and FL-AGS3. (**D**) ITC measurement of the interaction between LGN-GL1234 and FL-LGN. (**E**) Sequence alignment of the GL motifs of AGS3 and LGN. The red triangles indicate the conserved phenylalanines. (**F**) Schematic diagram of the domain organizations of FL-AGS3-4E and FL-LGN-4E. (**G**) ITC-based binding affinities of FL-AGS3-4E and FL-LGN-4E mutants to Insc (18–66) and NuMA (1886–1912). The raw data and fits are shown in Supplementary Figure S4.

LGN adopts an auto-inhibited conformation through intramolecular interactions between its TPR and GoLoco domains (Pan et al., 2013). In this study, we characterized the intramolecular interactions within AGS3. The results from ITC measurement revealed a binding affinity of 0.17 μM between AGS3-TPR and AGS3-GoLoco (Figure 2A). To examine the auto-inhibition, fusion proteins of AGS3 and LGN were constructed by replacing the linker sequences between the TPR and GoLoco domains with eight pairs of GS amino acids. The resultant fusion proteins were designated as FL-AGS3 and FL-LGN (Figure 2B). Subsequent ITC experiments demonstrated that both FL-AGS3 and FL-LGN lost the ability to bind to their isolated GoLoco domains (Figure 2C and D), indicating that they adopt an auto-inhibited conformation characterized by strong intramolecular interactions between the TPR and GoLoco domains.

The sequence and structure analysis of the GL motifs reveals a high level of conservation of key hydrophobic amino acids (F486, F487, F539, F591, and F624) essential for TPR interactions in both AGS3 and LGN (Pan et al., 2013; Figure 2E; Supplementary Figure S3). Based on this observation, we hypothesized that substitution of these Phe with charged amino acids in all four GL motifs could disrupt the auto-inhibition. Mutant proteins, where all four Phe residues were replaced by Glu, were designated as FL-AGS3-4E and FL-LGN-4E (Figure 2F). Subsequent ITC results indicated that both FL-AGS3-4E and FL-LGN-4E mutant proteins exhibited strong binding affinities with Insc or NuMA, similar to the affinities observed for the isolated TPR domain (Figure 2G; Supplementary Figure S4). These data suggest that, akin to LGN, the 4E mutation disrupts intramolecular interactions in AGS3, thereby releasing the auto-inhibited conformation.

### Both Insc and NuMA could open the auto-inhibited conformation of AGS3

To elucidate the influence of Insc and NuMA on the auto-inhibited conformation, we measured the binding affinities between Insc or NuMA and the FL-AGS3 or FL-LGN protein employing ITC. The results showed that Insc (18–66) or NuMA (1808–2001) could directly bind to FL-AGS3 and FL-LGN (Table 1; Supplementary Figure S5). Moreover, the binding affinities exhibited no substantial augmentation in the presence of Gα_i3_⋅GDP (Table 1; Supplementary Figure S5). This suggests that both Insc and NuMA have the ability to perturb the auto-inhibited conformation of AGS3, and Gα_i3_⋅GDP does not play a requisite role in facilitating the binding of Insc or NuMA with AGS3.

**Table 1:** **Binding affinities of FL-AGS3 and FL-LGN to their partners**.

**Partners**		***K*_D_ (μM)**
NuMA (1808–2001)	FL-AGS3	3.61 ± 0.10
	FL-AGS3 + Gα_i3_⋅GDP	2.28 ± 0.59
	FL-LGN	3.13 ± 0.37
	FL-LGN + Gα_i3_⋅GDP	2.24 ± 0.30
Insc (18–66)	FL-AGS3	2.27 ± 0.35
	FL-AGS3 + Gα_i3_⋅GDP	2.29 ± 0.31
	FL-LGN	3.92 ± 0.69
	FL-LGN + Gα_i3_⋅GDP	2.62 ± 0.48
Gα_i3_⋅GDP	FL-AGS3	1.60 ± 0.45
	FL-LGN	1.17 ± 0.13

We additionally characterized the interaction between Gα_i3_⋅GDP and FL-AGS3/FL-LGN. The results indicate that Gα_i3_⋅GDP binds to FL-AGS3 and FL-LGN with comparable affinities (Table 1; Supplementary Figure S6). These suggest that Gα_i3_⋅GDP is able to open the auto-inhibited conformations of AGS3 and LGN without the assistance of other proteins.

### Long-form AGS3-TPR with additional N-terminal residues could form oligomeric complexes with NuMA

**Figure 3:**
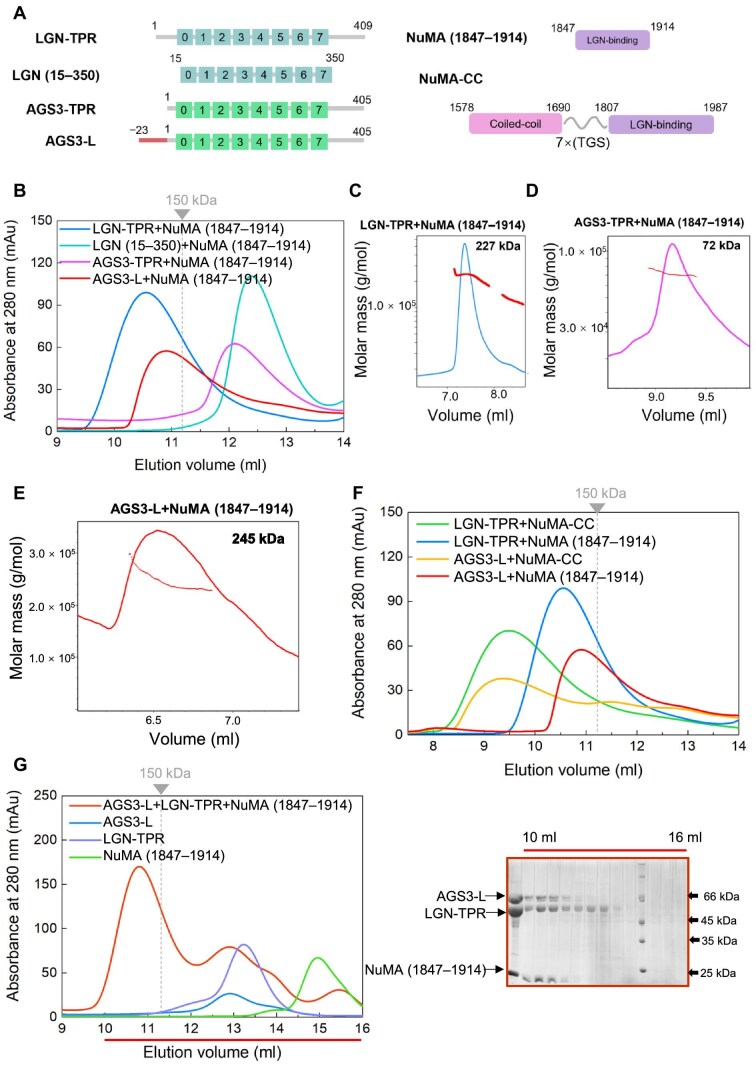
Long-form AGS3-TPR with additional N-terminal residues could form a multimeric complex with NuMA. (**A**) Schematic diagram of the domain organizations of various fragments of AGS3, LGN, and NuMA. (**B**) SEC analyses of the complexes formed between LGN-TPR, AGS3-TPR, or AGS3-L and NuMA (1847–1914). The dashed line indicates the elution volume of a 150-kDa globular protein marker. (**C**–**E**) SLS characterization of the MW of the LGN-TPR/NuMA (1847–1914) complex (**C**), AGS3-TPR/NuMA (1847–1914) complex (**D**), or AGS3-L/NuMA (1847–1914) complex (**E**). (**F**) SEC analyses of the complexes formed between LGN-TPR or AGS3-L and NuMA-CC. (**G**) SEC analyses of the complex formed between AGS3-L, LGN-TPR, and NuMA (1847–1914). Coomassie-stained SDS–PAGE gel to the right shows the protein composition of the elution profile.

Recent structural studies have revealed that LGN and NuMA form specific hetero-hexamers and also bind to dimeric full-length NuMA, thus generating an extended subcortical protein network (Pirovano et al., 2019). These oligomeric LGN/NuMA complexes facilitate microtubule capture at the cell cortex, essential for spindle orientation in epithelial cells (Pirovano et al., 2019). The ability of the TPR domain of LGN to form higher-order oligomeric complexes with NuMA appears to be critical for its spindle orientation function. However, it remains unclear whether AGS3 and NuMA can also form analogous oligomeric complexes. According to the literature findings, the shortest segments capable of forming hetero-hexamers are LGN (7–367) and NuMA (1847–1914) fragments (Pirovano et al., 2019). Consistent with these reports, our SEC results indicated that LGN-TPR (1–409)/NuMA (1847–1914) formed a complex (the peak at ∼10.5 ml) with a molecular weight (MW) well above 150 kDa, lager than that of the binary LGN (15–350)/NuMA (1847–1914) complex (the peak at ∼12.4 ml) (Figure 3A and B). Static light scattering (SLS) experiment further confirmed that the MW of the LGN-TPR/NuMA (1847–1914) complex was 227 kDa (Figure 3C), consistent with the formation of a 3:3 hetero-hexamer. Moreover, the shorter NuMA fragment, i.e. NuMA (1886–1912), in conjunction with LGN-TPR failed to form an oligomer with the MW higher than that of a 1:1 complex (Supplementary Figure S7A). These results highlight the necessity of the N-terminal 14 residues of LGN and the extended NuMA fragment (1847–1914) for the formation of LGN/NuMA hetero-hexamer.

In contrast, the SEC analysis of the AGS3-TPR/NuMA (1847–1914) mixture (the peak at ∼12.1 ml) exhibited an MW below 150 kDa, indicating the absence of an oligomeric complex (Figure 3B). Further SLS experiment confirmed the MW of AGS3-TPR/NuMA (1847–1914) as 72 kDa, consistent with the 1:1 binding ratio (Figure 3D). Sequence alignment of AGS3 and LGN reveals that AGS3-TPR (1–405) corresponds to LGN (13–409) (Supplementary Figure S7C), which lacks the critical N-terminal residues necessary for LGN hexamer formation. We hypothesized that a longer isoform with additional N-terminal residues might be required for AGS3 to form a multimeric complex with NuMA. Therefore, we purified a longer form of AGS3-TPR containing additional 23 residues at the N-terminus (Uniprot entry Q86YR5-1), denoted as AGS3-L (−23–405, with the N-terminal residues labeled from −23 to −1 to align with the sequence numbering of the short isoform) (Figure 3A), and then examined its mixture with NuMA (1847–1914).

AGS3-L exhibited similar binding affinities to NuMA and Insc compared to AGS3-TPR (Supplementary Figure S8). SEC analysis revealed that, similar to LGN-TPR, AGS3-L formed a complex with NuMA (1847–1914) (the peak at ∼10.9 ml) with an MW significantly exceeding 150 kDa (Figure 3B). SLS measurement further verified that the MW of the complex was 245 kDa (Figure 3E), indicative of a 3:3 hetero-hexamer complex. Additionally, when the NuMA fragment was truncated to (1886–1912), AGS3-L was no longer able to form a hexamer with NuMA (Supplementary Figure S7B). These findings underscore the requirement for the additional N-terminal residues in AGS3-L for efficient formation of a hetero-hexamer with NuMA.

To gain insights into the architecture of the AGS3-L/NuMA oligomeric complex, we built a structural model of the AGS3-L/NuMA (1847–1914) hexametric complex using homology modeling (Webb and Sali, 2016) based on the crystal structure of LGN/NuMA hexamer (PDB: 6HC2). The model reveals two critical structural characteristics necessary for hexamer formation (Supplementary Figure S9A). First, the additional 23 residues at the N-terminus of AGS3-L fold into a helix and form four-helix bundle with the TPR8 motif from the adjacent AGS3-L (Supplementary Figure S9B). Second, the N-terminal region of NuMA (1847–1914) interacts with the neighboring AGS3-L and contributes to stabilizing the hexamer structure (Supplementary Figure S9C). Overall, the structural model of AGS3-L/NuMA hexamer verifies the necessity of the N-terminal extension of the long-form AGS3 in forming the oligomeric complex with NuMA.

Full-length NuMA is known to form dimers through its coiled-coil domain, and previous studies have demonstrated that dimeric NuMA facilitates the formation of the higher-than-hexamer LGN/NuMA complex (Pirovano et al., 2019). To explore this further, we constructed a fusion protein connecting NuMA's coiled-coil domain (1578–1690) with its LGN-binding domain (1807–1987) via a 7(TGS) linker, designated as NuMA-CC (i.e. 1578–1690–7(TGS)–1807–1987) (Figure 3A). In agreement with the previous study (Pirovano et al., 2019), SEC analysis showed that the size of the LGN-TPR/NuMA-CC complex (the peak at ∼9.5 ml) was significantly larger than that of LGN-TPR/NuMA (1847–1914) (Figure 3F), suggesting the formation of a higher-order multimeric complex beyond a hexamer. Similarly, SEC analysis indicated that the MW of the complex formed by AGS3-L and NuMA-CC (the peak at ∼9.3 ml) was also much higher than that of AGS3-L/NuMA (1847–1914) (Figure 3F), indicating that AGS3-L can form a high-order hetero-oligomer with NuMA-CC as well. These results underscore the capacity of both LGN and AGS3 to engage in higher-order complex formation with NuMA.

### Competitive binding between AGS3 and LGN for NuMA

Subsequently, we investigated the competitive binding between AGS3 and LGN for NuMA. A mixture containing AGS3-L, LGN-TPR, and NuMA (1847–1914) in the 1:1:1 ratio was analyzed using SEC to assess the composition of the complex formed (the peak at ∼10.8 ml) (Figure 3G). The results confirmed the presence of all three components, indicating that both LGN and AGS3 can engage with NuMA (Figure 3G). Furthermore, a GST pull-down experiment was conducted to evaluate the binding of AGS3-L and LGN-TPR to NuMA (1808–2001) at various concentration ratios. The results demonstrated that AGS3-L and LGN-TPR displayed comparable binding abilities for NuMA (Supplementary Figure S10). Moreover, an excess of AGS3-L over LGN-TPR resulted in the displacement of LGN-TPR and complete occupancy of NuMA (Supplementary Figure S10A). Conversely, an excessive amount of LGN-TPR predominantly led to the formation of the LGN-TPR/NuMA complex (Supplementary Figure S10B). These findings highlight the competitive nature of AGS3 and LGN for binding to NuMA, influenced by their relative concentrations.

### Full-length AGS3 and LGN have lower capacity to form oligomeric complexes with NuMA

**Figure 4:**
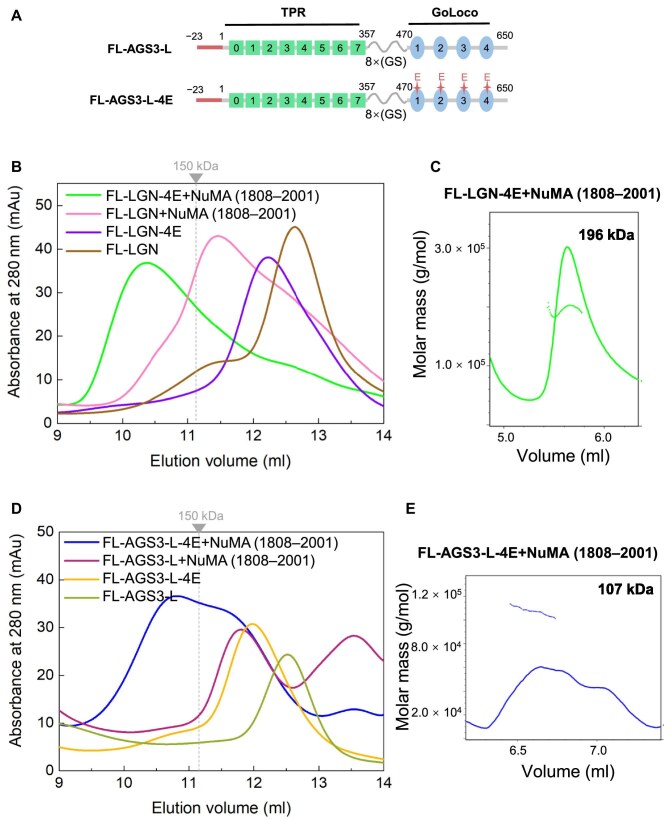
Full-length LGN and AGS3 have lower capacity to form oligomeric complexes with NuMA. (**A**) Schematic diagram of the domain organizations of long isoforms FL-AGS3-L and FL-AGS3-L-4E. (**B**) SEC analyses of the complexes formed between FL-LGN or FL-LGN-4E and NuMA (1808–2001). The dashed line indicates the elution volume of a 150-kDa globular protein marker. (**C**) SLS characterization of the MW of the FL-LGN-4E/NuMA (1808–2001) complex. (**D**) SEC analyses of the complexes formed between FL-AGS3-L or FL-AGS3-L-4E and NuMA (1808–2001). (**E**) SLS characterization of the MW of the FL-AGS3-L-4E/NuMA (1808–2001) complex.

Both LGN and AGS3 exhibit strong intramolecular interactions in their full-length forms, which may impact the assembly of high-order oligomers with NuMA. In order to investigate the binding of full-length LGN and AGS3 to NuMA, we utilized the FL-LGN fusion proteins mentioned earlier and engineered a long-form AGS3 protein, which includes the N-terminal 23 residues (i.e. −23–357–8(GS)–470–650), designated as FL-AGS3-L (Figure 4A). Additionally, the preceding data indicated that NuMA (1808–2001) can bind to FL-LGN and FL-AGS3 in the absence of Gα_i3_⋅GDP. Therefore, we conducted SEC analysis to examine the mixture of FL-LGN or FL-AGS3-L with NuMA (1808–2001). The results showed that FL-LGN forms a 1:1 complex with NuMA (1808–2001) (the peak at ∼11.5 ml) (Figure 4B). This contrasts with LGN-TPR, which forms a 3:3 hetero-hexamer with NuMA (Figure 3B), suggesting that intramolecular interactions within FL-LGN hinder the formation of a multimeric complex. Introducing the 4E mutation in FL-LGN to disrupt these intramolecular interactions, the resultant FL-LGN-4E protein leads to the formation of a complex with the higher MW (the peak at ∼10.3 ml) when mixed with NuMA (1808–2001) (Figure 4A). The SLS experiment revealed that the MW of the FL-LGN-4E/NuMA (1808–2001) complex was 196 kDa, indicative of a 2:2 hetero-tetramer (Figure 4C). These results suggest that only in the open conformational state can the FL-LGN form a multimeric complex with NuMA, and the GoLoco domain impedes the formation of high-order oligomer even without intramolecular interactions.

For AGS3, the SEC profile demonstrated that the FL-AGS3-L fusion protein forms a 1:1 complex with NuMA (1808–2001) (the peak at ∼11.8 ml) (Figure 4D). Upon introducing the FL-AGS3-L-4E mutant to disrupt the intramolecular interaction, the elution volume of complex shifted to a smaller volume (the peak at ∼10.8 ml) (Figure 4D). However, the peak was broad, and the MW was obviously lower compared to that of the FL-LGN-4E/NuMA complex (Figure 4D). Subsequent SLS experiment indicated an MW of 107 kDa, implying primarily a 1:1 complex (Figure 4E). Consequently, these results indicate that FL-AGS3-L is unable to form a stable multimeric complex with NuMA, even in the open conformation. This suggests that AGS3 has a lower capacity than LGN to engage in high-order complex formation with NuMA.

### The impact of Insc on the multimeric complex formed between LGN or AGS3 and NuMA

**Figure 5:**
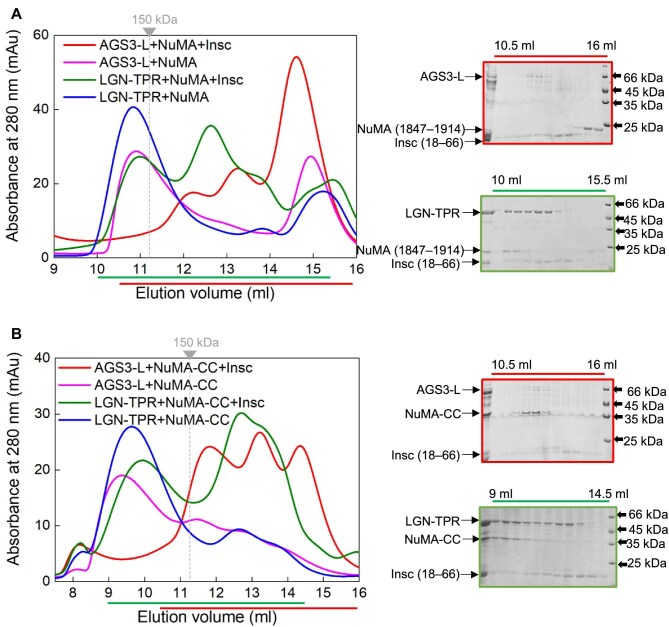
The impact of Insc on the oligomeric complexes formed between LGN or AGS3 and NuMA. (**A**) SEC analyses of the complexes formed between LGN-TPR or AGS3-L and NuMA (1847–1914) in the presence or absence of Insc (18–66). Coomassie-stained SDS–PAGE gels to the right show the protein composition of two elution profiles. (**B**) SEC analyses of the complexes formed between LGN-TPR or AGS3-L and NuMA-CC in the presence or absence of Insc (18–66). Coomassie-stained SDS–PAGE gels to the right show the protein composition of two elution profiles.

We have demonstrated that the TPR domains of both AGS3 and LGN exhibit a preference for binding with Insc over NuMA. The preceding findings suggest that LGN-TPR and AGS3-L can form multimeric complexes with NuMA. These observations prompted us to investigate whether the TPR domain of AGS3 or LGN retains this preference in the context of multimeric complex formation with NuMA. To address this question, LGN-TPR or AGS3-L proteins were mixed with either NuMA (1847–1914) or NuMA-CC along with Insc (18–66) protein in a 1:2:2 ratio, and the resulting mixtures were subsequently analyzed by SEC. The results revealed that the introduction of Insc leads to a slight reduction in the quantity of LGN/NuMA hetero-hexamer complexes (the peak at ∼10.8 ml) (Figure 5A), indicating that Insc partially disrupts the LGN/NuMA hetero-hexamers. This suggests that Insc competes, to some extent, with NuMA (1847–1914) for binding to LGN. Similarly, the high-order LGN-TPR/NuMA-CC complex (the peak at ∼9.5 ml) was also partly disrupted by the addition of Insc (18–66) (Figure 5B), albeit to a lesser degree compared to NuMA (1847–1914) (Figure 5A).

In contrast, the multimeric complexes AGS3-L/NuMA (1847–1914) (the peak at ∼10.9 ml) and AGS3-L/NuMA-CC (the peak at ∼9.3 ml) were nearly completely disrupted by Insc (18–66) (Figure 5A and B). A significant portion of AGS3-L instead formed a complex with Insc (18–66) (the peak detected at ∼12.1 ml) (Figure 5A and B). These findings suggest that the high-order oligomeric complexes formed between AGS3-L and NuMA are less stable than those between LGN and NuMA. Therefore, these results support the conclusion that Insc maintains a preference for binding to the TPR domain of AGS3, even in the context of oligomeric complex formation with NuMA.

### N-terminal sequences of AGS3 and LGN determine their different capacities in forming oligomeric complexes with NuMA

**Figure 6:**
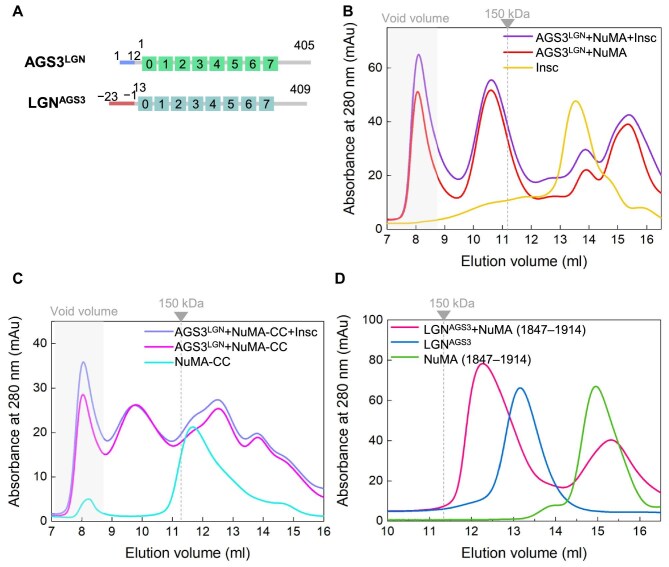
Different N-terminal sequences determine different capacities of AGS3 and LGN in forming multimeric complexes with NuMA. (**A**) Schematic diagram of the domain organizations of chimeric proteins AGS3^LGN^ and LGN^AGS3^. (**B**) SEC analyses of the complexes formed between AGS3^LGN^ and NuMA (1847–1914) in the presence or absence of Insc (18–66). The gray box shows the void volume. (**C**) SEC analyses of the complexes formed between AGS3^LGN^ and NuMA-CC in the presence or absence of Insc (18–66). (**D**) SEC analyses of the complex formed between LGN^AGS3^ and NuMA (1847–1914).

We hypothesized that the reduced stability of the oligomeric AGS3-L/NuMA complexes might be attributed to the less conserved N-terminal residues in the AGS3-L sequence, which exhibit lower homology with the corresponding region of the LGN (Supplementary Figure S7C). To investigate the significance of these N-terminal sequences in LGN and AGS3 for their ability to oligomerize with NuMA, we engineered a chimeric fusion protein. This construct substituted the N-terminal segment (−23∼−1) of AGS3-L with residues 1–12 from the LGN, resulting in the construct AGS3^LGN^, i.e. LGN (1–12)–AGS3 (1–405) (Figure 6A). In a reciprocal approach, we also generated an LGN-TPR fusion protein, LGN^AGS3^, i.e. AGS3 (−23∼–1)–LGN (13–409), where the N-terminal 1–12 amino acids of LGN were replaced with the N-terminal segment (−23∼−1) from AGS3 (Figure 6A).

Subsequent SEC analyses were conducted to examine the interactions between AGS3^LGN^ or LGN^AGS3^ and NuMA (1847–1914). The results demonstrated that AGS3^LGN^ exhibited the ability to form a stable multimeric complex with NuMA (1847–1914), and the presence of Insc did not disrupt this interaction (Figure 6B). This behavior markedly contrasts with AGS3-L, which failed to maintain a stable hetero-hexamer complex with NuMA in the presence of Insc (Figure 5A). Similarly, for the AGS3^LGN^/NuMA-CC complex, the addition of Insc also produced negligible interference effects (Figure 6C).

Conversely, unlike LGN-TPR, the LGN^AGS3^ protein was incapable of forming a hetero-hexamer complex with NuMA, instead forming a 1:1 complex (Figure 6D). These experimental findings reinforce the notion that the non-conserved N-terminal residues preceding the TPR motifs in LGN and AGS3 play a critical role in determining their ability to form a high-order multimeric complex with NuMA.

### AGS3-L S−2Y mutant enhances hydrophobic interaction to form stable hexamers with NuMA

**Figure 7:**
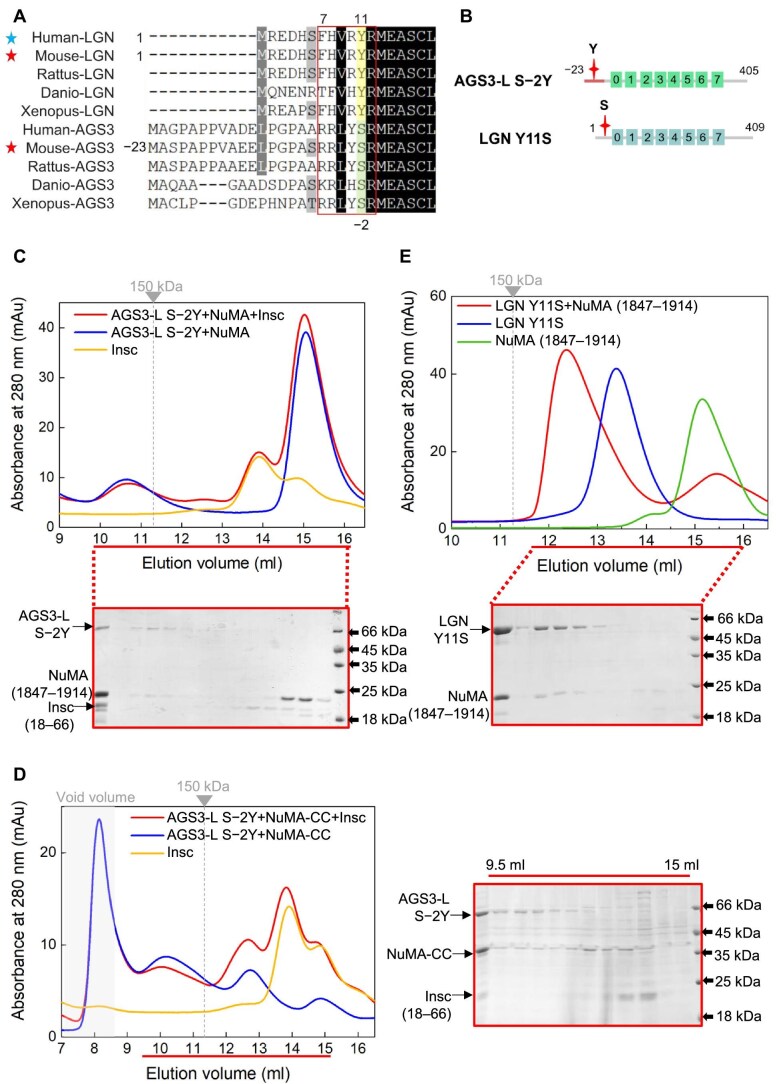
AGS3-L S−2Y mutant enhances hydrophobic interaction, facilitating the formation of a stable hexamer with NuMA. (**A**) Multisequence alignment of N-terminal sequences of LGN and AGS3 from five species, including human, mouse, *Rattus norvegicus, Danio rerio*, and *Xenopus tropicalis*. The red stars indicate the specific sequences of LGN and AGS3 utilized in this study, while the blue star denotes the sequence of LGN in the crystal structure of the LGN/NuMA hexamer (PDB: 6HC2). (**B**) Schematic representation of the domains of the mutant proteins AGS3-L S−2Y and LGN Y11S. (**C**) SEC analyses of the complexes formed between AGS3-L S−2Y and NuMA (1847–1914) in the presence or absence of Insc (18–66). (**D**) SEC analyses of the complexes formed between AGS3-L S−2Y and NuMA-CC in the presence or absence of Insc (18–66). The gray box denotes the void volume. (**E**) SEC analyses of the complex formed between LGN Y11S and NuMA (1847–1914). Coomassie-stained SDS–PAGE gels show the protein composition of the corresponding elution profiles.

The results indicate that the 12 residues at the N-terminal of LGN are essential for the formation of stable LGN-TPR/NuMA hexamers. To further investigate this, we conducted a comparative analysis of the conservation of these residues within the AGS3-L N-terminal sequence, aiming to identify the key residues that significantly contribute to the stability of AGS3-L/NuMA hexamers. The N-terminal sequences of LGN and AGS3-L show considerable divergence (Figure 7A). However, previous studies have demonstrated that the LGN fragment comprising residues 7–367 is the shortest capable of forming a hexamer with NuMA (1847–1914). Based on this, we hypothesize that the key residue contributing to the functional difference between AGS3 and LGN likely resides within residues 7–12 of LGN (^7^FHVRYR^12^), while the corresponding sequence in AGS3-L spans residues −6 to −1 (^−6^RRLYSR^−1^) (Figure 7A). To pinpoint this key residue, we compared the crystal structure of the LGN/NuMA hexamer (PDB: 6HC2) with a structural model of the AGS3-L/NuMA hexamer predicted through homology modeling (Supplementary Figure S11). Notably, the side chain of Tyr11 in one LGN monomer (LGN-2) forms hydrophobic interactions with the side chain of Leu359 in the adjacent LGN monomer (LGN-1, Supplementary Figure S11). In contrast, the corresponding Ser−2 residue in AGS3-L (AGS3-2 monomer) does not establish a similar hydrophobic interaction with the Met370 residue on the adjacent monomer (AGS3-1, Supplementary Figure S11).

Based on these observations, we introduced targeted mutations at the N-terminus of AGS3-L and LGN, specifically the S−2Y mutation in AGS3-L and the Y11S mutation in LGN (Figure 7B). We then examined the interaction between the mutant proteins and NuMA (1847–1914) using SEC analysis. The results revealed that the mixture of the AGS3-L S−2Y mutant and NuMA (the peak at 10.8 ml) displayed an MW exceeding 150 kDa, indicating the formation of a 3:3 hexameric complex (Figure 7C). Notably, the introduction of Insc did not significantly affect the stability of this hexameric complex (Figure 7C), in stark contrast to the wild-type AGS3-L (Figure 5A). Similarly, the higher-order multimeric complex formed between AGS3-L S−2Y and NuMA-CC remained stable in the presence of Insc (Figure 7D). On the other hand, the LGN Y11S mutant, unlike wild-type LGN-TPR (Figure 3B), failed to form a hexamer with NuMA, with a binding ratio of 1:1 (Figure 7E). These results suggest that Tyr11 at the N-terminus of LGN is critical for hexamer formation, a residue replaced by Ser in AGS3-L. Sequence alignment also shows that the Tyr11 residue is conserved in LGN orthologues (Figure 7A).

## Discussion

**Figure 8:**
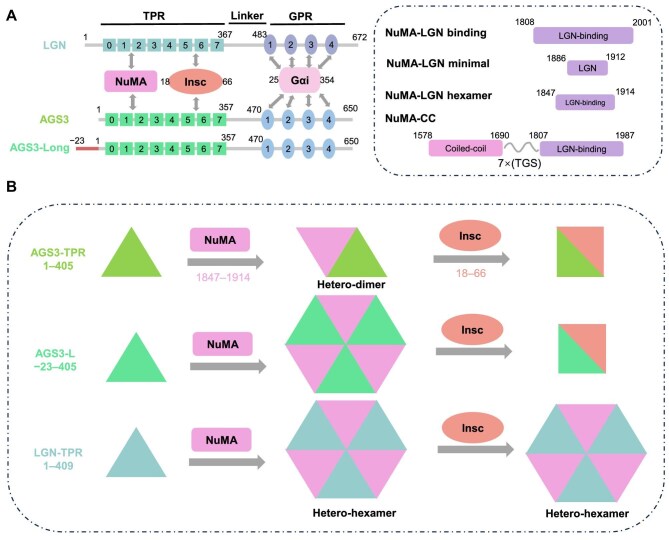
A model illustrating the molecular mechanism underlying the distinct roles of LGN and AGS3 in regulating oriented cell divisions. (**A**) The paralogues LGN and AGS3 share a high degree of sequence similarity and exhibit similar characteristics in their interactions with NuMA and Insc. (**B**) The multimeric AGS3/NuMA complex exhibits reduced stability compared to the LGN/NuMA complex and is more susceptible to disruption by Insc.

In this research, we conducted a systematic examination aimed at discerning the structural and functional disparities between AGS3 and LGN, with a particular emphasis on biochemical analysis of the intra- and intermolecular interactions of the TPR and GoLoco domains. Our findings revealed that the isolated TPR or GoLoco domains of the both proteins exhibit comparable binding affinities toward their respective partners, which include NuMA, Insc, and Gα_i3_⋅GDP. Minimal variations were observed in intramolecular interactions and in the release of their auto-inhibited conformations. The primary distinction observed between AGS3 and LGN lies in their respective abilities to form oligomeric complexes with NuMA. The short-form AGS3-TPR without the N-terminal 23 residues displayed a capacity to solely form a 1:1 complex with NuMA, whereas the long-form AGS3-TPR also presented a weaker propensity compared to LGN to form a high-order oligomeric complex with NuMA. Notably, Insc was capable of disrupting the oligomeric AGS3/NuMA complex, while the LGN/NuMA hexamer complex remained stable even in the presence of Insc (Figure 8). These *in vitro* findings provide insights into the molecular mechanism underlying the distinct roles of LGN and AGS3 in regulating oriented cell divisions.

Our study corroborated previous observations that AGS3, due to its inability to form stable high-order oligomeric complexes with NuMA, cannot effectively regulate spindle orientation (Pirovano et al., 2019). This aligns with experimental evidence demonstrating that AGS3 could not rescue spindle orientation defects in LGN knockdown cells (Saadaoui et al., 2017). Importantly, our findings demonstrated that both AGS3-TPR and FL-AGS3 exhibit binding affinity to NuMA comparable to those of LGN (Table 1; Supplementary Figure S8). Additionally, we demonstrated that AGS3 can compete with LGN for binding to NuMA (Supplementary Figure S10). This competitive binding suggests that AGS3 plays a significant regulatory role in modulating the dynamics of the LGN/NuMA complex.

Consequently, we propose that AGS3 most likely plays a role in reducing the abundance of the oligomeric LGN/NuMA complex that is crucial for proper spindle orientation. These finding support the observation that AGS3 functions antagonistically to LGN in inhibiting the perpendicular divisions (Descovich et al., 2023). *In vivo* and cell biology studies have shown that the precise regulation of spindle orientation is essential for cell fate determination and tissue architecture. By elucidating the competitive interaction between AGS3 and LGN for NuMA binding, our study enhances the understanding of the molecular mechanisms governing spindle orientation.

It is noteworthy that both full-length LGN and AGS3 exhibit a significantly reduced capacity to form oligomeric complexes with NuMA, even in the open conformation induced by the 4E mutation. The binding affinity to NuMA seems unaffected by Gα_i3_·GDP, suggesting that Gα_i3_·GDP is unlikely to facilitate the release of the auto-inhibited conformation. Another significant regulatory factor influencing the formation of oligomeric complexes between LGN or AGS3 and NuMA is Insc, which has the ability to disrupt the hetero-hexamer complex of AGS3-L/NuMA. Insc is known to localize to the apical cortex during ACD (Kamakura et al., 2013). Although AGS3 has limited ability to form an oligomeric complex with NuMA, Insc may also play an additional role in hindering the formation of a functional complex between AGS3 and NuMA for spindle orientation. Furthermore, Insc may exhibit a preference for binding to AGS3 over LGN, given that the oligomeric LGN/NuMA complex is resistant to Insc binding. As a result, AGS3 could potentially compete with LGN for Insc binding, and effectively sequestering LGN away from the apical cortex. This aligns with the observation that overexpression of AGS3 could weaken the polarized distribution of LGN (Descovich et al., 2023).

In the present study, we did not identify any functional disparities between the GoLoco domains of AGS3 and LGN *in vitro*. The individual GL motifs, the entire GoLoco domains, and the full-length proteins of AGS3 and LGN all demonstrate analogous binding affinities to Gα_i3_·GDP. These results suggest that AGS3 and LGN likely possess comparable affinities toward Gα_i_  *in vivo* as well. Consequently, the distinctive cellular localization patterns observed for AGS3 and LGN are unlikely to be primarily attributable to differences in their interactions with Gα_i_. It is conceivable that additional proteins may play a role in regulating the localization dynamics of LGN and AGS3 within a cell. Further investigation into these additional regulatory factors could provide deeper insights into the mechanisms governing the spatial distribution and functional activities of LGN and AGS3 in cellular contexts.

Overall, these insights underscore the functional significance of AGS3 as a modulator of spindle orientation, suggesting that it may contribute to the regulation of cell division and, consequently, developmental processes.

## Materials and methods

### Protein expression and purification

The TPR domains of mouse LGN (1–409) and mouse AGS3 (1–405 or −23–405), the GoLoco domains of LGN (483–650) and AGS3 (470–631), and the N-terminal fragment of mouse Insc (18–66), as well as the C-terminal fragments of human NuMA (1886–1912 or 1808–2001), were individually inserted into a modified version of the pET32a vector, pET28a-sumo, or pGEX-6P-1 vector. Consequently, the resultant proteins featured a trx tag, sumo tag, or GST tag at the N-termini. Human Gα_i3_ was cloned into a pET-M3C vector, resulting in a protein with a His6 tag at the N-termini. The FL-AGS3 and FL-LGN fusion proteins were cloned into a modified version of pET32a vector. All mutations were generated through the standard polymerase chain reaction-based mutagenesis method and confirmed via DNA sequencing. Subsequently, the recombinant proteins were expressed in *Escherichia coli* BL21 (DE3) host cells at 16°C and purified using Ni^2+^-NTA or GST-agarose affinity chromatography followed by SEC.

### GST pull-down assay

GST or GST-tagged proteins (final concentration: 4 μM) were initially applied to 50 μl GSH-Sepharose 4B slurry beads in 500 μl assay buffer containing 50 mM Tris (pH 8.0), 100 mM NaCl, 1 mM 2-mercaptoethanol, and 1 mM ethylenediaminetetraacetic acid (EDTA). Subsequent to four times of buffer washing, the beads loaded with GST fusion proteins were mixed with potential binding partners (final concentration: 8 μM), and the mixtures were incubated for 1 h at 4°C. After four additional washing steps, proteins captured by the affinity beads were eluted by boiling, separated via 12% SDS–PAGE, and visualized through Coomassie blue staining.

### ITC

ITC measurements were performed using a Malvern PEAQ-ITC Micro calorimeter (Micro Cal) at 25°C. All protein samples were dissolved in a buffer containing 50 mM Tris (pH 8.0), 100 mM NaCl, and 1 mM EDTA. For AGS3-TPR or AGS3-L protein, the salt concentration of NaCl is increased to 300 mM for optimal solubility. Titrations were carried out by injecting 40-μl aliquots of one protein (0.2 mM) into another protein (0.02 mM). The injections were spaced at 1- or 2-min intervals to ensure that the titration peak returned to the baseline. The titration data were analyzed using the Origin software provided by Microcal and fitted with the one-site binding model.

### Analytical SEC

An AKTA FPLC system (GE Healthcare) was employed to conduct analytical SEC. Protein samples were applied onto a Superose 12 10/300 GL column (GE Healthcare), which had been pre-equilibrated with a buffer containing 50 mM Tris–HCl (pH 8.0), 100 mM NaCl, 1 mM 2-mercaptoethanol, and 1 mM EDTA. The concentrations of AGS3-TPR, AGS3-L, LGN-TPR, and related AGS3 or LGN proteins were set at 10 μM. The concentrations of NuMA (1847–1914), NuMA (1808–2001), NuMA-CC, and Insc were in excessive, at 20 μM, corresponding to a 1:2:2 ratio.

### SLS

Protein characterization was accomplished through SEC coupled to multi-angle light scattering (MALS). MALS was conducted using the HELEOS II instrument from Wyatt Technology. Generally, 100 μl of concentrated purified samples, at an approximate concentration of 10 μM, were applied to the columns.

## Supplementary Material

mjae049_Supplemental_File

## References

[bib1] Adhikari A., Sprang S.R. (2003). Thermodynamic characterization of the binding of activator of G protein signaling 3 (AGS3) and peptides derived from AGS3 with Gα. J. Biol. Chem. 278, 51825–51832. 14530282 10.1074/jbc.M306300200

[bib2] Bernard M.L., Peterson Y.K., Chung P. et al. (2001). Selective interaction of AGS3 with G-proteins and the influence of AGS3 on the activation state of G-proteins. J. Biol. Chem. 276, 1585–1593. 11042168 10.1074/jbc.M005291200

[bib3] Blumer J.B., Lord K., Saunders T.L. et al. (2008). Activator of G protein signaling 3 null mice: I. Unexpected alterations in metabolic and cardiovascular function. Endocrinology 149, 3842–3849. 18450958 10.1210/en.2008-0050 PMC2488243

[bib4] Blumer J.B., Oner S.S., Lanier S.M. (2012). Group II activators of G-protein signalling and proteins containing a G-protein regulatory motif. Acta Physiol. 204, 202–218. 10.1111/j.1748-1716.2011.02327.x 21615707

[bib5] Bowers M.S., Hopf F.W., Chou J.K. et al. (2008). Nucleus accumbens AGS3 expression drives ethanol seeking through Gβγ. Proc. Natl Acad. Sci. USA 105, 12533–12538. 18719114 10.1073/pnas.0706999105 PMC2527946

[bib6] Culurgioni S., Alfieri A., Pendolino V. et al. (2011). Inscuteable and NuMA proteins bind competitively to Leu–Gly–Asn repeat-enriched protein (LGN) during asymmetric cell divisions. Proc. Natl Acad. Sci. USA 108, 20998–21003. 22171003 10.1073/pnas.1113077108 PMC3248549

[bib7] Descovich C.P., Lough K.J., Jena A. et al. (2023). AGS3 antagonizes LGN to balance oriented cell divisions and cell fate choices in mammalian epidermis. eLife 12, e80403. 37017303 10.7554/eLife.80403 PMC10115442

[bib8] Du Q., Macara I.G. (2004). Mammalian Pins is a conformational switch that links NuMA to heterotrimeric G proteins. Cell 119, 503–516. 15537540 10.1016/j.cell.2004.10.028

[bib9] Fan P., Jiang Z., Diamond I. et al. (2009). Up-regulation of AGS3 during morphine withdrawal promotes cAMP superactivation via adenylyl cyclase 5 and 7 in rat nucleus accumbens/striatal neurons. Mol. Pharmacol. 76, 526–533. 19549762 10.1124/mol.109.057802 PMC2730385

[bib10] Groves B., Gong Q., Xu Z. et al. (2007). A specific role of AGS3 in the surface expression of plasma membrane proteins. Proc. Natl Acad. Sci. USA 104, 18103–18108. 17991770 10.1073/pnas.0709282104 PMC2084303

[bib11] Izaki T., Kamakura S., Kohjima M. et al. (2006). Two forms of human inscuteable-related protein that links Par3 to the Pins homologues LGN and AGS3. Biochem. Biophys. Res. Commun. 341, 1001–1006. 16458856 10.1016/j.bbrc.2006.01.050

[bib12] Jia M., Li J., Zhu J. et al. (2012). Crystal structures of the scaffolding protein LGN reveal the general mechanism by which GoLoco binding motifs inhibit the release of GDP from Gα_i_. J. Biol. Chem. 287, 36766–36776. 22952234 10.1074/jbc.M112.391607 PMC3481280

[bib13] Kamakura S., Nomura M., Hayase J. et al. (2013). The cell polarity protein mInsc regulates neutrophil chemotaxis via a noncanonical G protein signaling pathway. Dev. Cell 26, 292–302. 23891662 10.1016/j.devcel.2013.06.008

[bib14] Kiyomitsu T., Cheeseman I.M. (2013). Cortical dynein and asymmetric membrane elongation coordinately position the spindle in anaphase. Cell 154, 391–402. 23870127 10.1016/j.cell.2013.06.010 PMC4177044

[bib15] Konno D., Shioi G., Shitamukai A. et al. (2008a). Neuroepithelial progenitors undergo LGN-dependent planar divisions to maintain self-renewability during mammalian neurogenesis. Nat. Cell Biol. 10, 93–101. 18084280 10.1038/ncb1673

[bib16] Konno D., Shioi G., Shitamukai A. et al. (2008b). Neuroepithelial progenitors undergo LGN-dependent planar divisions to maintain self-renewability during mammalian neurogenesis. Nat. Cell Biol. 10, 93–101. 18084280 10.1038/ncb1673

[bib17] Lechler T., Mapelli M. (2021). Spindle positioning and its impact on vertebrate tissue architecture and cell fate. Nat. Rev. Mol. Cell Biol. 22, 691–708. 34158639 10.1038/s41580-021-00384-4 PMC10544824

[bib18] Morrison S.J., Kimble J. (2006). Asymmetric and symmetric stem-cell divisions in development and cancer. Nature 441, 1068–1074. 16810241 10.1038/nature04956

[bib19] Pan Z., Zhu J., Shang Y. et al. (2013). An autoinhibited conformation of LGN reveals a distinct interaction mode between GoLoco motifs and TPR motifs. Structure 21, 1007–1017. 23665171 10.1016/j.str.2013.04.005

[bib20] Pattingre S., De Vries L., Bauvy C. et al. (2003). The G-protein regulator AGS3 controls an early event during macroautophagy in human intestinal HT-29 cells. J. Biol. Chem. 278, 20995–21002. 12642577 10.1074/jbc.M300917200

[bib21] Pirovano L., Culurgioni S., Carminati M. et al. (2019). Hexameric NuMA:LGN structures promote multivalent interactions required for planar epithelial divisions. Nat. Commun. 10, 2208. 31101817 10.1038/s41467-019-09999-w PMC6525239

[bib22] Regner K.R., Nozu K., Lanier S.M. et al. (2011). Loss of activator of G-protein signaling 3 impairs renal tubular regeneration following acute kidney injury in rodents. FASEB J. 25, 1844–1855. 21343176 10.1096/fj.10-169797 PMC3101034

[bib23] Saadaoui M., Konno D., Loulier K. et al. (2017). Loss of the canonical spindle orientation function in the Pins/LGN homolog AGS3. EMBO Rep. 18, 1509–1520. 28684399 10.15252/embr.201643048 PMC5579374

[bib24] Sanada K., Tsai L.H. (2005). G protein βγ subunits and AGS3 control spindle orientation and asymmetric cell fate of cerebral cortical progenitors. Cell 122, 119–131. 16009138 10.1016/j.cell.2005.05.009

[bib25] Wavreil F.D.M., Yajima M. (2020). Diversity of activator of G-protein signaling (AGS)-family proteins and their impact on asymmetric cell division across taxa. Dev. Biol. 465, 89–99. 32687894 10.1016/j.ydbio.2020.07.004 PMC7484151

[bib26] Webb B., Sali A. (2016). Comparative protein structure modeling using MODELLER. Curr. Protoc. Bioinformatics 54, 5.6.1–5.6.37. 10.1002/cpbi.3 PMC5031415 27322406

[bib27] Williams S.E., Ratliff L.A., Postiglione M.P. et al. (2014). Par3-mInsc and Gα_i3_ cooperate to promote oriented epidermal cell divisions through LGN. Nat. Cell Biol. 16, 758–769. 25016959 10.1038/ncb3001 PMC4159251

[bib28] Wu P.S., Egger B., Brand A.H. (2008). Asymmetric stem cell division: lessons from Drosophila. Semin. Cell Dev. Biol. 19, 283–293. 18328747 10.1016/j.semcdb.2008.01.007

[bib29] Yan J., Zhang K., Guo T. et al. (2022). Par3 regulates the asymmetric division of basal stem cells in psoriasis via the Par3/mInsc/LGN signaling axis. Cell. Immunol. 373, 104496. 35259602 10.1016/j.cellimm.2022.104496

[bib30] Yip J.L.K., Lee M.M.K., Leung C.C.Y. et al. (2020). AGS3 and Gα_i3_ are concomitantly upregulated as part of the spindle orientation complex during differentiation of human neural progenitor cells. Molecules 25, 5169. 33172018 10.3390/molecules25215169 PMC7664263

[bib31] Yuzawa S., Kamakura S., Iwakiri Y. et al. (2011). Structural basis for interaction between the conserved cell polarity proteins inscuteable and Leu–Gly–Asn repeat-enriched protein (LGN). Proc. Natl Acad. Sci. USA 108, 19210–19215. 22074847 10.1073/pnas.1110951108 PMC3228485

[bib32] Zheng Z., Wan Q., Liu J. et al. (2013). Evidence for dynein and astral microtubule-mediated cortical release and transport of Gα_i_/LGN/NuMA complex in mitotic cells. Mol. Biol. Cell 24, 901–913. 23389635 10.1091/mbc.E12-06-0458 PMC3608500

[bib33] Zhu J., Wen W., Zheng Z. et al. (2011). LGN/mInsc and LGN/NuMA complex structures suggest distinct functions in asymmetric cell division for the Par3/mInsc/LGN and Gαi/LGN/NuMA pathways. Mol. Cell 43, 418–431. 21816348 10.1016/j.molcel.2011.07.011 PMC3158460

