# Supplementary material for: Molecular insights into AGS3’s role in spindle orientation: a biochemical perspective
Source: J Mol Cell Biol. 2024 Nov 23;16(11):mjae049. doi: 10.1093/jmcb/mjae049 (PMC12151148; doi:10.1093/jmcb/mjae049)
Supplement: mjae049_Supplemental_File [file mjae049_supplemental_file.pdf]

Supplementary material

Molecular Insights into AGS3's Role in Spindle Orientation: A Biochemical Perspective

Shi Yu[1], Jie Ji[1], Jingwei Weng[1], Zhijun Liu[2, *], Wenning Wang[1, *]

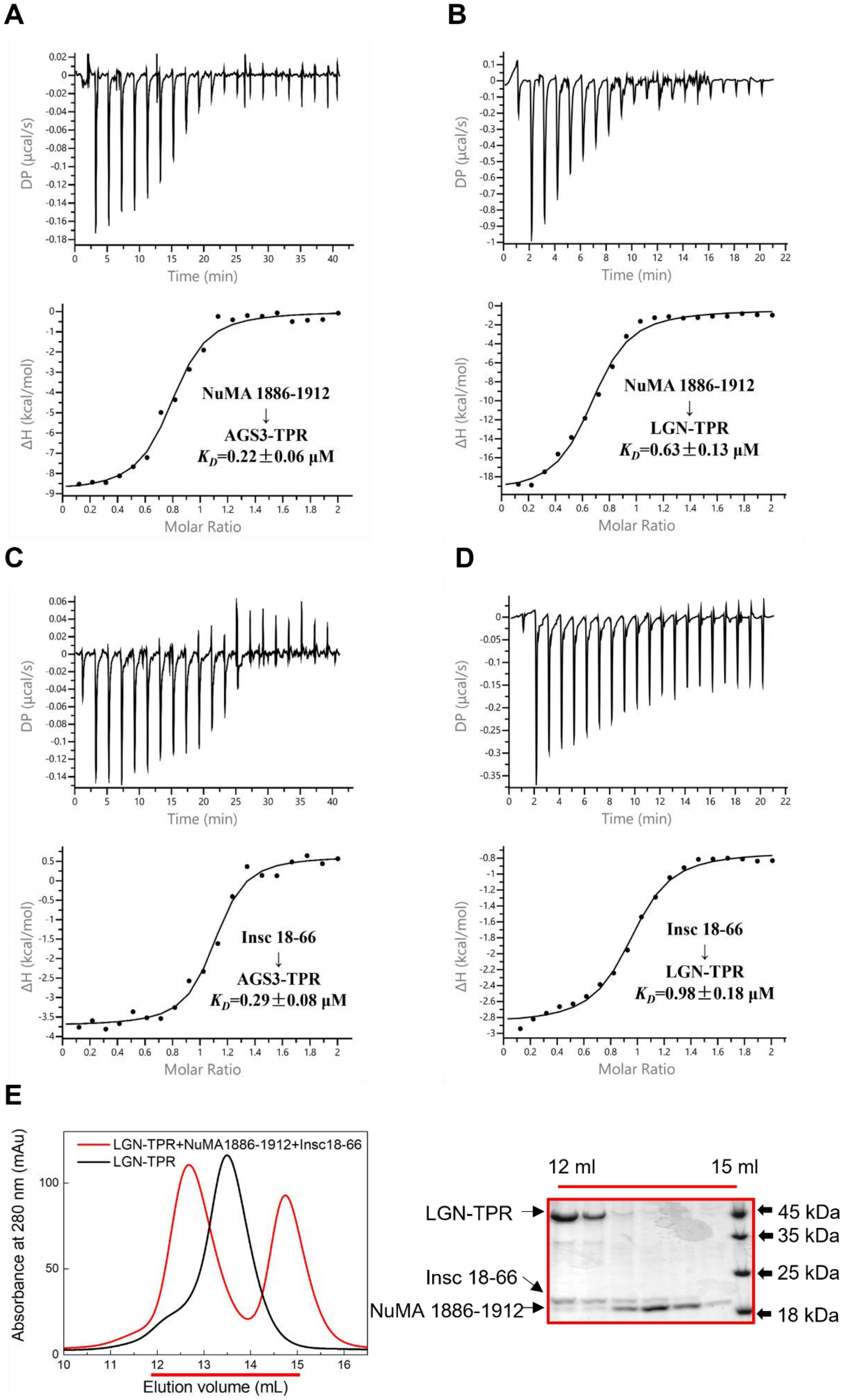

Figure S1. AGS3-TPR binds to Insc and NuMA similarly to LGN-TPR. (A-D) ITC

measurements of the bindings of AGS3-TPR/LGN-TPR protein to NuMA (1886–1912) and Insc (18–66). (E) The SEC analysis of the mixture of LGN-TPR, NuMA (1886–1912) and Insc (18–66) at a 1:2:2 ratio.

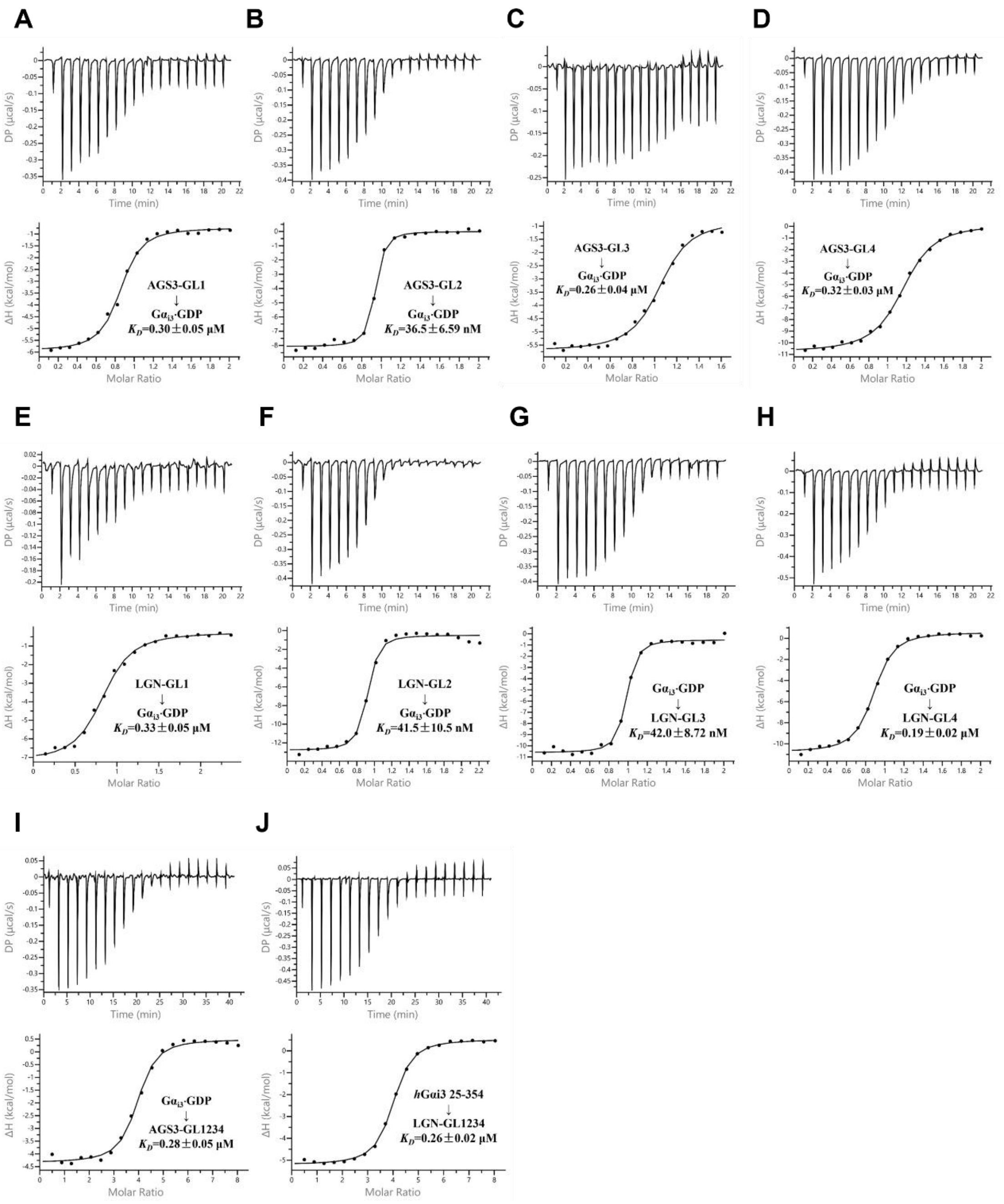

Figure S2. (A–D) ITC measurements of the bindings of $G\alpha_{i3}\cdot$GDP to AGS3 GL motifs. (E–H) ITC measurements for the binding affinities of $G\alpha_{i3}\cdot$GDP to LGN GL motifs. (I–J) ITC measurements for the binding affinities of $G\alpha_{i3}\cdot$GDP to the GoLoco domains of AGS3 and LGN.

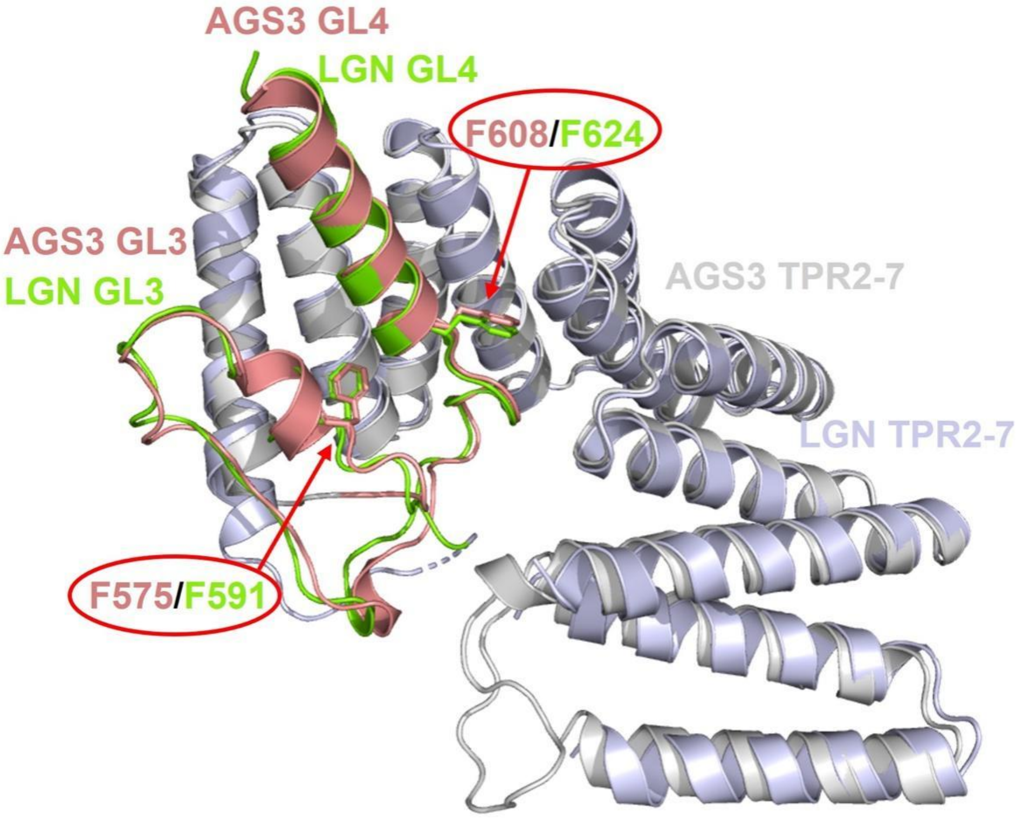

Figure S3. Comparison of the crystal structure of LGN TPR2–7-GL34 (PDB:4JHR) with the predicted structure model of AGS3 TPR2–7-GL34 generated by homology modelling. The LGN GL34 sequence is depicted in green, while the AGS3 GL34 sequence is shown in salmon. The corresponding conserved phenylalanine residues in the GL3 and GL4 motifs are illustrated as sticks.

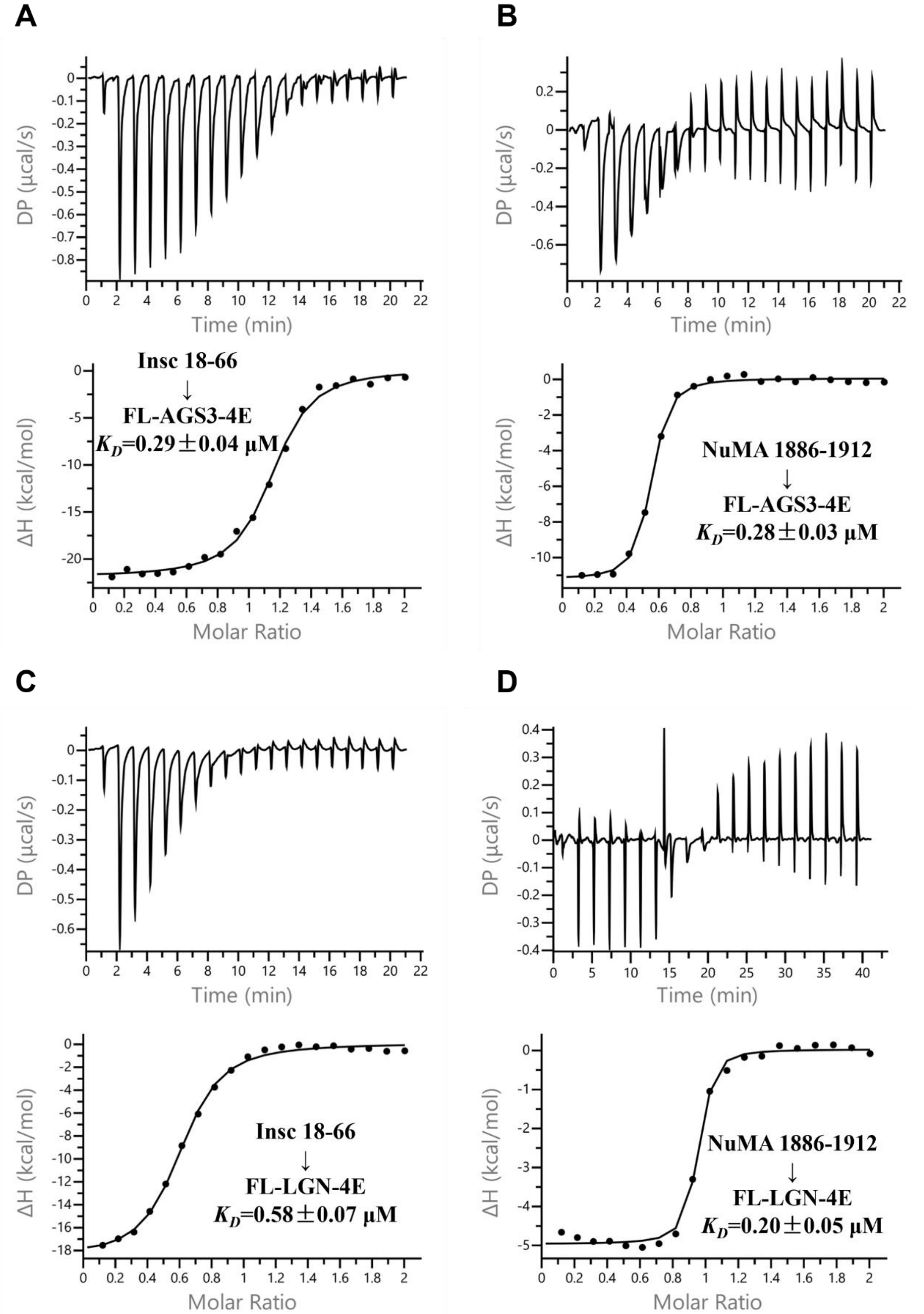

Figure S4. ITC measurements of the bindings of FL-AGS3-4E or FL-LGN-4E protein to Insc (18–66) and NuMA (1886–1912).

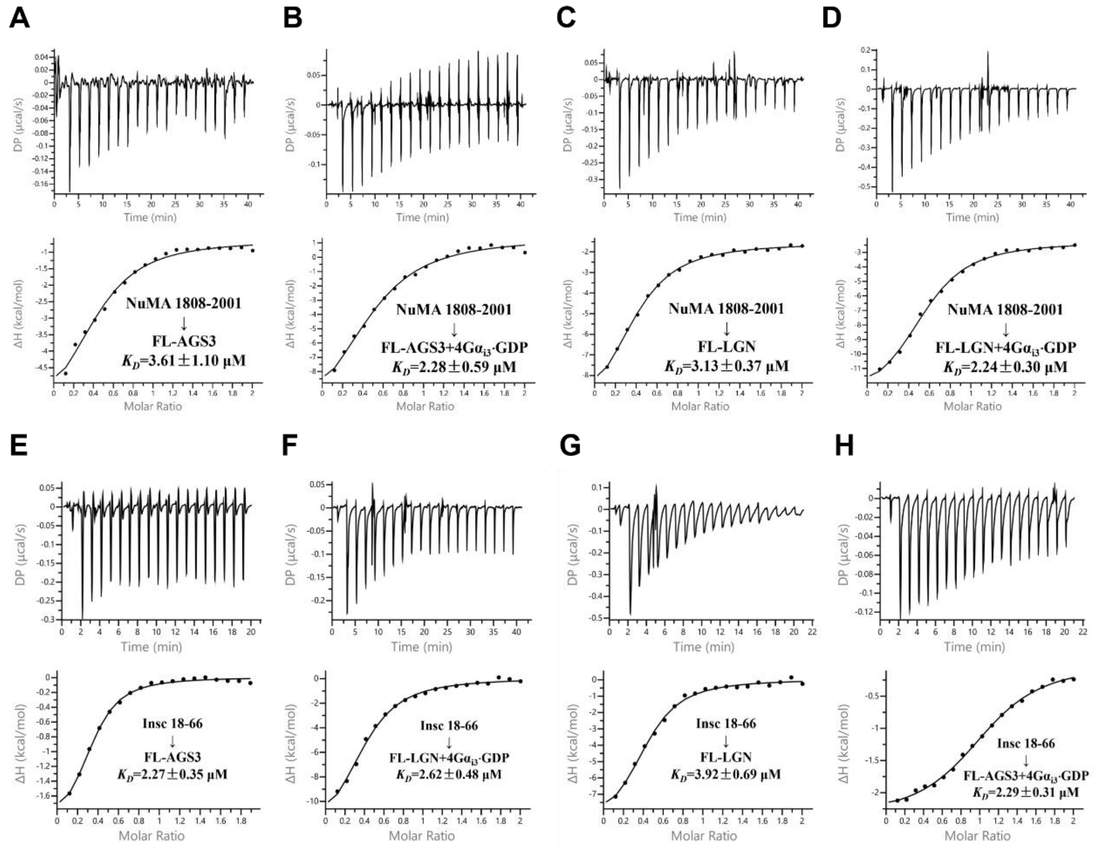

Figure S5. (A–D) The ITC measurements of the interactions between NuMA (1808–2001) and FL-AGS3/FL-LGN protein in the presence or absence of Gα_{i3}·GDP. (E–H) The ITC measurements of the interactions between Insc (18–66) and FL-AGS3/FL-LGN in the presence or absence of Gα_{i3}·GDP.

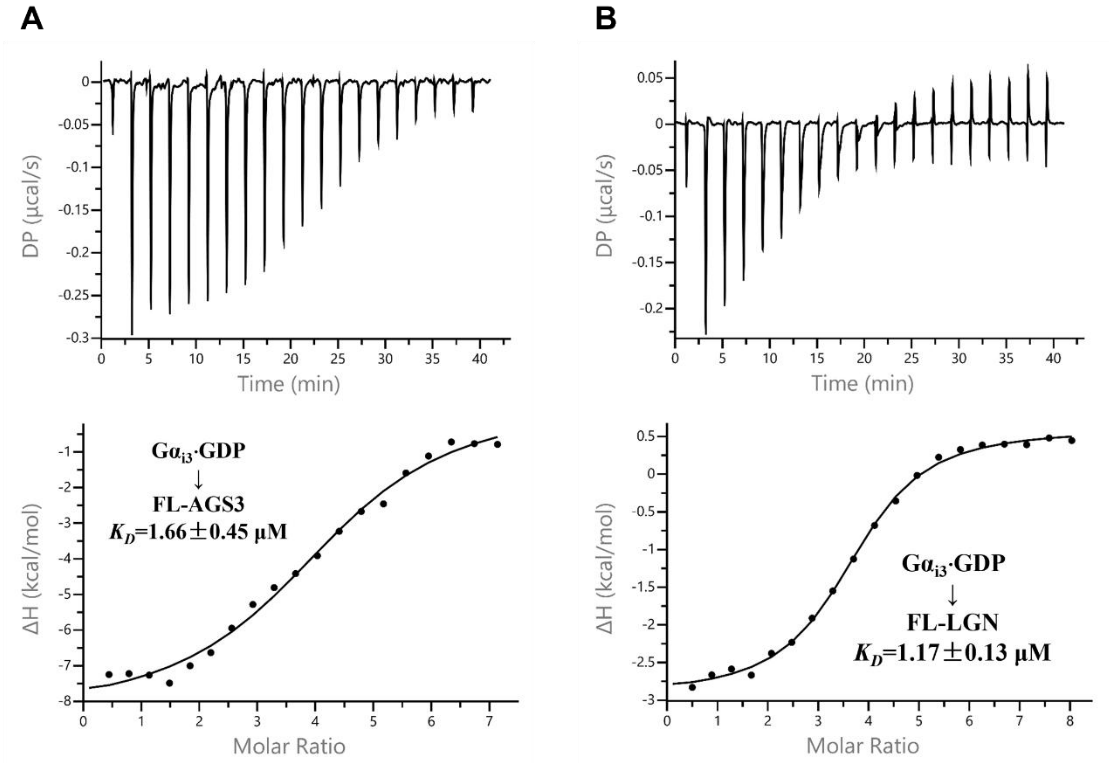

Figure S6. The ITC measurements for the interactions between $G\alpha_{i3} \cdot GDP$ and FL-AGS3 (A) or FL-LGN (B).

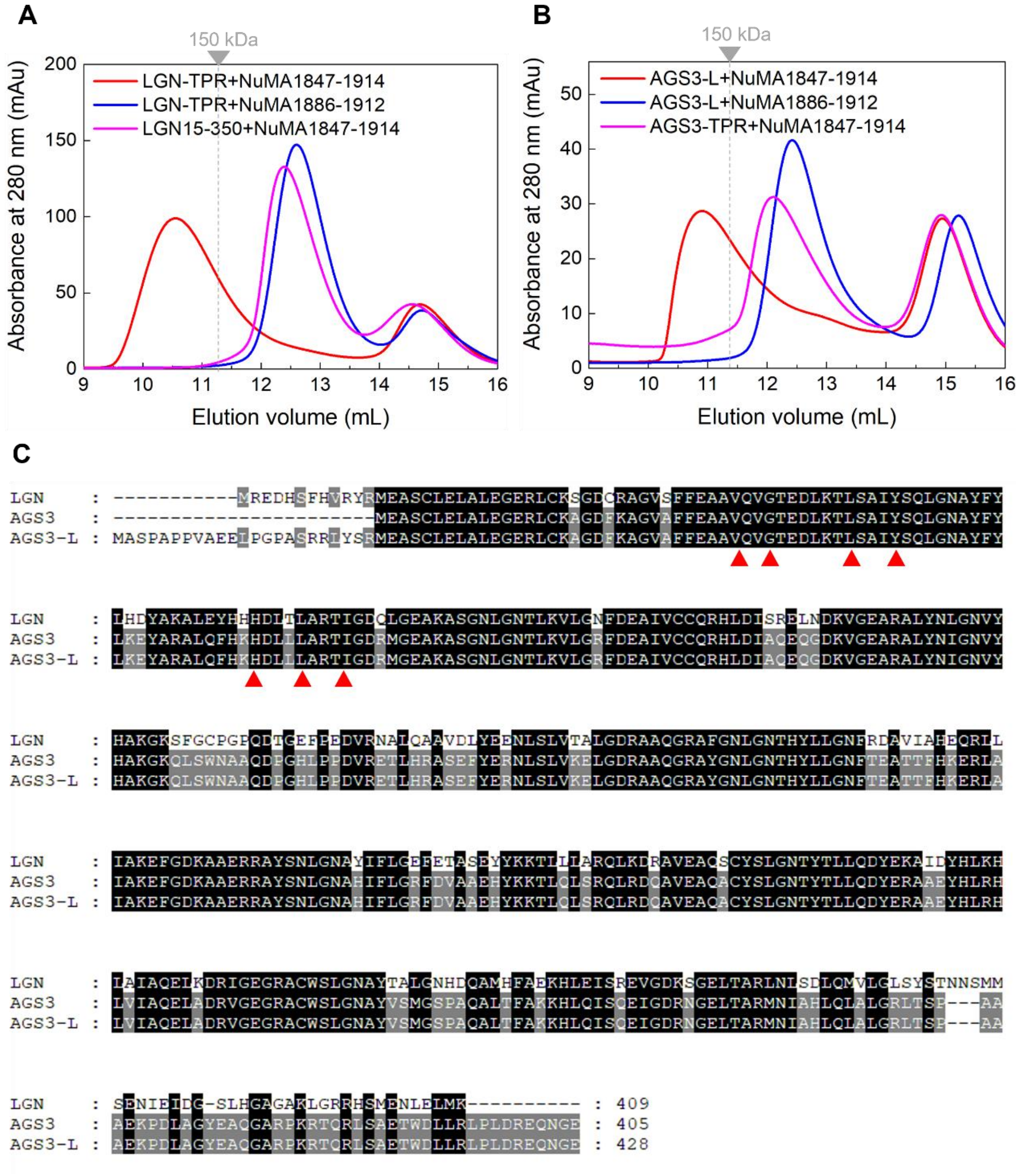

Figure S7. (A) SEC analyses of the complexes formed between various fragments of LGN-TPR and NuMA (1847–1914) or NuMA (1886–1912). (B) SEC analyses of the complexes formed between various fragments of AGS3-TPR and NuMA (1847–1914) or NuMA (1886–1912). (C) Sequence alignment of the N-terminal regions of LGN and AGS3 isoforms. The long isoform AGS3-L has additional 23 residues in the N-terminal which shows low homology comparing to LGN. The red triangles indicate the conserved residues in TPR that interact with NuMA (1847–1861).

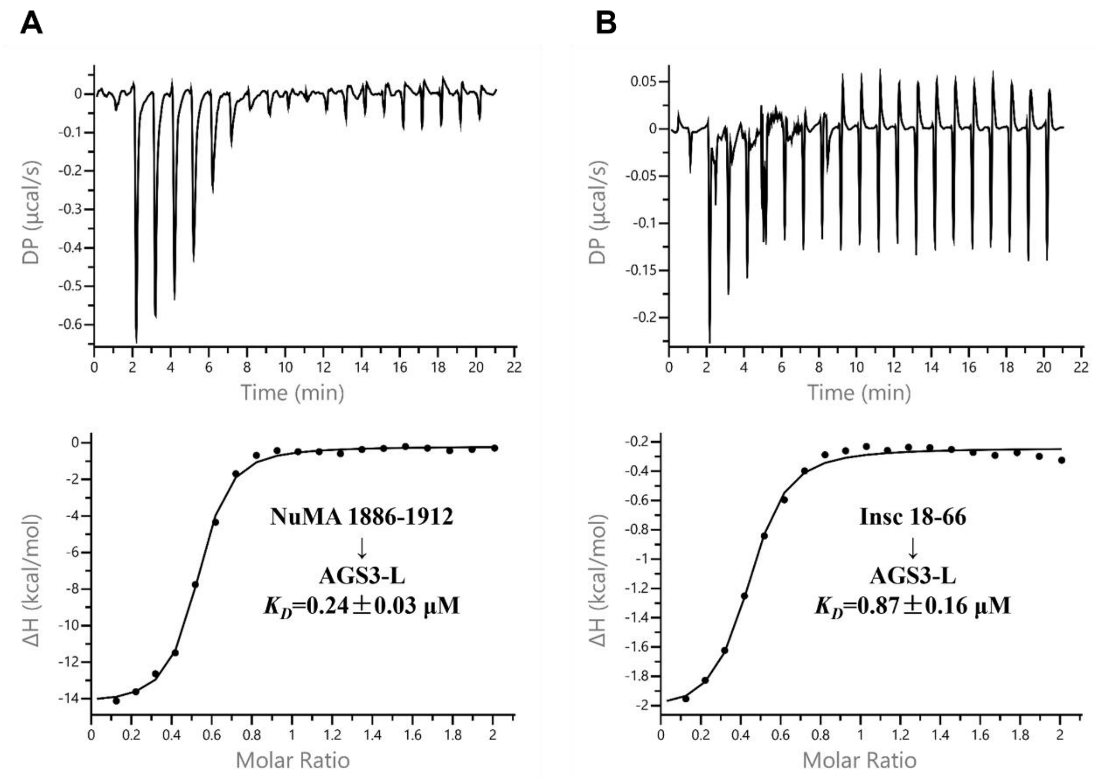

Figure S8. ITC measurements for the interactions between AGS3-L and NuMA (1886–1912) (A) or Insc (18–66) (B).

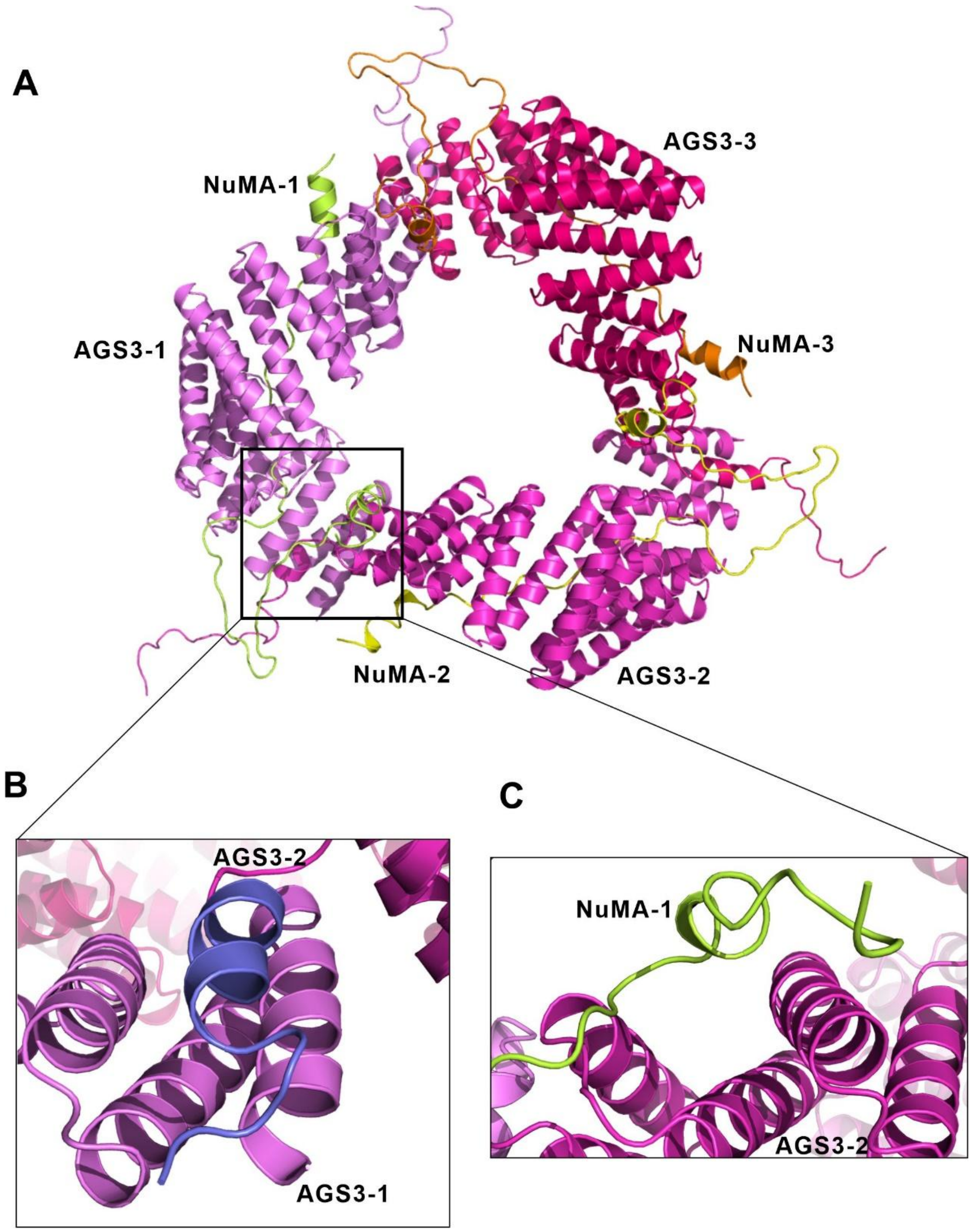

Figure S9. (A) The structural model of AGS3-L/NuMA hexamer complex. (B) The N-terminal of AGS3-L interacts with the adjacent AGS3 TPR8 to form a four-helix bundle. The −23 to −1 residues of AGS3-1 are highlighted in slate blue. (C) The N-terminal part of NuMA-1 interacts with the neighboring AGS3-2 to facilitate the hexamer formation.

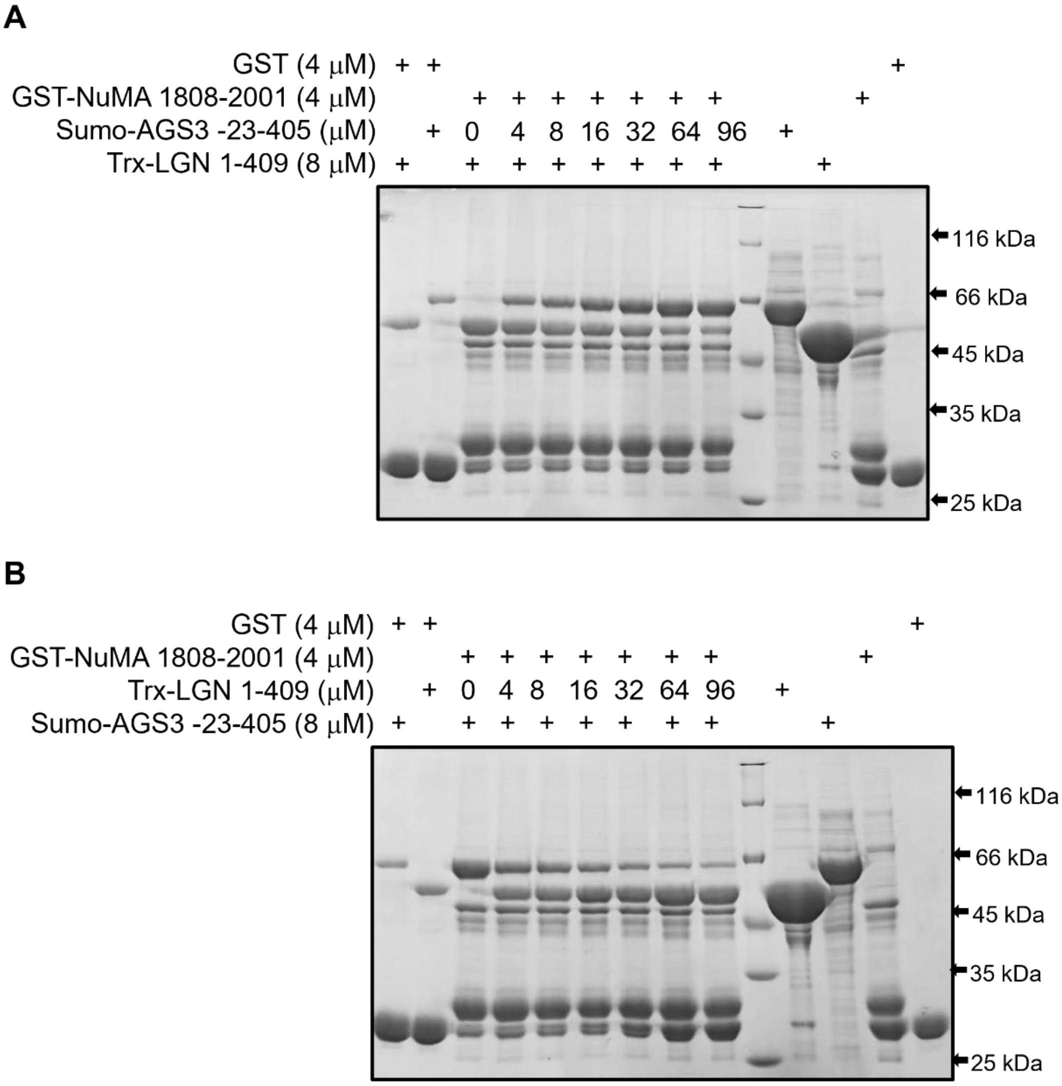

Figure S10. (A) GST Pull-down assay shows that with the increase of AGS3-L, the amount of NuMA-bound LGN-TPR decreased. (B) GST Pull-down assay shows that with the increase of LGN-TPR, the amount of NuMA-bound AGS3-L decreased.

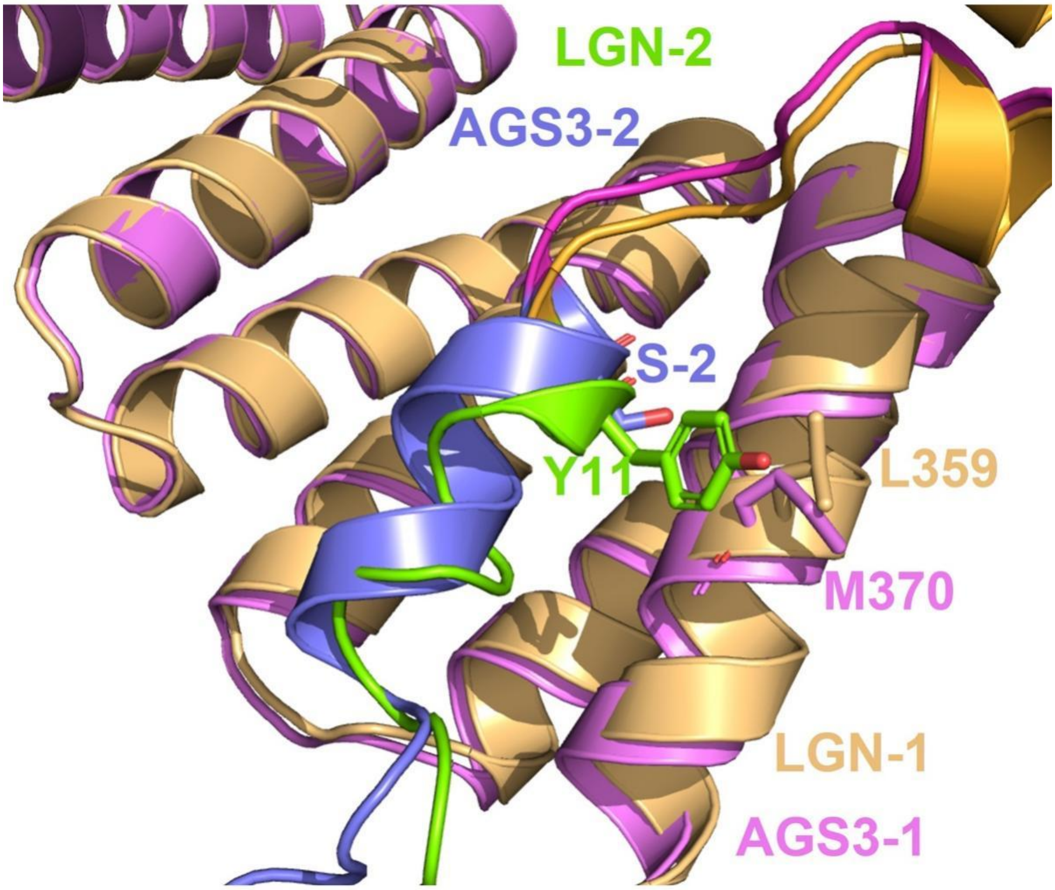

Figure S11. Interaction of the AGS3-L N-terminal sequence with the adjacent TPR8 motif, highlighting the absence of hydrophobic interaction between residue Y11 in the LGN-TPR and residue L359 in the adjacent TPR.
